# Exploring the relationship between self-employment and women’s cardiovascular health

**DOI:** 10.1186/s12905-022-01893-w

**Published:** 2022-07-23

**Authors:** Sedina Dzodzomenyo, Kimberly Danae Cauley Narain

**Affiliations:** 1 UCLA David Geffen School of Medicine, University of California, Los Angeles, 10833 Le Conte Ave, Los Angeles, CA 90095 USA; 2 Division of General Internal Medicine and Health Services Research (GIM/HSR), Department of Medicine, University of California, Los Angeles, 1100 Glendon Ave., Suite 850, Los Angeles, CA 90024 USA; 3 Center for Health Advancement, Fielding School of Public Health, University of California, Los Angeles, Box 951772, 650 Charles Young Dr. S. 31-269 CHS, Los Angeles, CA 90095 USA

**Keywords:** Cardiovascular disease, Women’s health, Employment status

## Abstract

**Background:**

Compared with wage and salary work, self-employment has been linked to more favorable cardiovascular health outcomes within the general population. Women comprise a significant proportion of the self-employed workforce and are disproportionately affected by cardiovascular disease. Self-employed women represent a unique population in that their cardiovascular health outcomes may be related to gender-specific advantages of non-traditional employment. To date, no studies have comprehensively explored the association between self-employment and risk factors for cardiovascular disease among women.

**Methods:**

We conducted a weighted cross-sectional analysis using data from the University of Michigan Health and Retirement Study (HRS). Our study sample consisted of 4624 working women (employed for wages and self-employed) enrolled in the 2016 HRS cohort. Multivariable linear and logistic regression were used to examine the relationship between self-employment and several self-reported physical and mental health risk factors for cardiovascular disease, controlling for healthcare access.

**Results:**

Among working women, self-employment was associated with a 34% decrease in the odds of reporting obesity, a 43% decrease in the odds of reporting hypertension, a 30% decrease in the odds of reporting diabetes, and a 68% increase in the odds of reporting participation in at least twice-weekly physical activity (*p* < 0.05). BMI for self-employed women was on average 1.79 units lower than it was for women working for wages (*p* < 0.01).

**Conclusions:**

Employment structure may have important implications for cardiovascular health among women, and future studies should explore the causal relationship between self-employment and cardiovascular health outcomes in this population.

*Trial Registration*: Not applicable.

## Background

Approximately 45% of women in the United States are living with some form of cardiovascular disease (CVD) [defined here as hypertension, coronary heart disease, stroke, or heart failure] [1]. In 2017, female deaths attributable to cardiovascular complications totaled 418,665, continuing the decades-long trend of CVD as the leading cause of death among women [1]. Despite the high burden of CVD, there remain significant disparities in the diagnosis, treatment, and outcomes of heart disease in women when compared to men. Women are more likely to be under- or mis-diagnosed and receive less guidance on CVD prevention measures [2]. This may be related to overdiagnosis of non-cardiac conditions (e.g., anxiety) in women, differences in cardiovascular disease presentation between women and men, and beliefs among providers about the incidence and prevalence of CVD in women [3]. Once a diagnosis has been made, women are prescribed statins at lower rates and are less likely to be referred for cardiac rehabilitation [2]. Again, this may be the result of lack of knowledge about the impact of CVD in women [4]. Consequently, women suffer higher rates of mortality following myocardial infarction relative to men [5].

Gender disparities in CVD outcomes are in part rooted in differences in risk factors for cardiovascular disease. Traditional cardiovascular risk factors including obesity, physical inactivity, and dyslipidemia are more prevalent among women, particularly in the postmenopausal period [2, 6]. In addition, many women experience female-specific risk factors such as polycystic ovarian syndrome and hypertensive disorders of pregnancy [7]. Finally, less well-known risk factors such as depression are more frequently associated with women than with men [2]. Importantly, psychosocial stress has been strongly linked with cardiovascular disease and has also consistently been shown to affect women at higher rates [8]. A recent study posits that up to 50% of female CVD patients experience myocardial ischemia induced specifically by mental stress [8].

The traditional workplace presents a unique set of stressors for women due in large part to adherence to the ideal worker model within United States workplaces, work-family conflict—the resolution of which continues to rest on the shoulders of women—and persistent gender discrimination [9, 10]. These factors likely have a significant impact on mental and physical health outcomes, including cardiovascular risk factors and outcomes [10]. Self-employment may reduce women’s exposure to gendered work stressors, which is particularly significant given the relationship between stress and cardiovascular health outcomes in women.

Previous research has established a positive correlation between self-employment and improved cardiovascular health among the general population. A 2013 study found that, compared to salary work, self-employment was positively associated with perceived physical health and negatively associated with hypertension, hyperlipidemia, diabetes, and stroke [11]. Self-employment has also been linked to reduced reports of “no exercise” and increased fruit and vegetable consumption among black women [12]. The relationship between self-employment and mental health is less well-studied, and available data are inconsistent [11, 12].

The observed improvements in cardiovascular health among self-employed individuals may be related to both direct and indirect health benefits stemming from increased work autonomy and greater schedule flexibility [11]. Evidence suggests that both autonomy and flexibility have favorable implications for mental health and stress. Autonomy is an important predictor of job satisfaction, which is positively associated with better mental health outcomes [13, 14]. Work autonomy has also been linked to reduced stress [15] In a similar vein, schedule flexibility has been associated with reduced “work-home conflict” (interference of work responsibilities with domestic commitments), high levels of which have been linked to mental stress among working women in particular [16]. These findings are meaningful given that stress levels have direct implications for cardiovascular disease risk factors such as hypertension. Indirect impacts of autonomy and flexibility on cardiovascular disease risk factors center around health behavior. Greater work autonomy and the ensuing reduced stress may decrease engagement in destructive health behaviors such as binge drinking and smoking [8]. Furthermore, schedule flexibility may allow time for higher engagement in health-promoting behaviors such as exercise, maintaining a healthy diet, and participation in healthcare maintenance visits [11].

In addition to increased autonomy and flexibility, self-employment may afford women the particularly valuable benefit of reduced exposure to workplace gender discrimination. Gendered perceptions of women (particularly those who choose to become parents) as less capable employees—and the consequent limitations on career advancement—have been posited as a motivating factor for pursuing self-employment [17]. Within traditional employment structures, women are subject to more unfavorable evaluations and fewer promotions relative to men, in addition to interpersonal mistreatment [17]. Reduced exposure to such discrimination may have positive health impacts given that workplace discrimination is associated with long-term emotional distress and functional limitations [18].

Although there is promising data on the relationship between self-employment and health outcomes within the general population, no studies to date have comprehensively explored the association between self-employment and cardiovascular disease risk factors with specific regard to women’s health. Given the existing knowledge of the link between employment structure and CVD risk factors and the disproportionate burden of such risk factors among women, it is important to examine the relationship between self-employment and women’s cardiovascular disease risk factors.

We conducted a weighted cross-sectional analysis using data from the University of Michigan Health and Retirement Study to investigate the association between self-employment and several health outcomes and health behaviors in women. Additionally, we ran models to determine if any of the observed relationships were impacted by access to healthcare. Healthcare access was considered because some studies have suggested that self-employment may be associated with limited access to health care [11]. Specifically, health insurance premiums in the individual market may be particularly steep, forcing self-employed individuals to forego health insurance coverage or purchase less generous insurance plans [11]. Consequently, high out-of-pocket healthcare costs may present a barrier to maintaining cardiovascular disease risk factor control among self-employed women. This may make self-employment a less realistic option for women with both suboptimal health and limited financial resources, leading to overrepresentation of relatively healthier, wealthier women within the female self-employed labor force.

We hypothesized a negative association between self-employment and cardiovascular disease risk factors. We also hypothesized that this relationship would not be driven by access to health care. Instead, we believed the relationship between self-employment and cardiovascular disease risk factors may be mediated primarily through effects of self-employment on autonomy, flexibility and gender-based discrimination.

## Methods

### Data source

We used data from the University of Michigan Health and Retirement Study (HRS), a longitudinal panel study comprised of questionnaire responses from approximately 20,000 adults across the United States. The HRS is supported by the National Institute on Aging and the Social Security Administration and aims to shed light on the health, family, and economic experiences of Americans over the age of 50. It is a rich data source containing detailed information on health status and associated risk factors, employment status and structure, healthcare access, and other variables capable of serving as proxies for resources that may be associated with both self-employment and health, such as neighborhood quality. The study employs a steady-state model in which a new, age-eligible cohort is added to the existing study sample every 6 years. There are currently seven sub-samples within the overall study sample (HRS, AHEAD, CODA, WAR BABY, EARLY BABY BOOMER, MIDDLE BABY BOOMER, and LATE BABY BOOMER). Data collection began in 1992, with respondents surveyed at 2-year intervals until death. The Early Baby Boomer and Mid Baby Boomer sub-samples were supplemented in the 2010 wave with a sample of individuals residing in areas with 10% or higher concentrations of Black and/or Hispanic populations in order to boost the size of the minority samples in those cohorts. Our data source was the 2016 HRS cohort, whose questionnaire responses were collected from April 2016–April 2018. In 2016, the American Diabetes Association (ADA) recommended screening for all adults age 45 and older for diabetes, irrespective of weight status [19]. Consequently, the 2016 HRS cohort includes a sizable proportion of women that would have been screened by their physicians for cardiovascular disease risk factor control, relative to earlier HRS cohorts.

### Participants and procedures

Of the 20,912 participants in the 2016 cohort, 12,242 were women. We were interested in the relationship between women’s health outcomes and employment structure and thus included in our analysis only those women who reported being employed at the time of data collection (N = 4624). Both self-employed women and women working for wages qualified. We chose to utilize a cross-sectional research design so our findings would not be confounded by the 2016 changes in the cardiovascular disease risk factor screening guidelines.

### Measures

All measures were obtained from the HRS. Our measures for employment structure were constructed from responses to the survey question, “On your current (main) job, do you work for someone else, are you self-employed, or what?” Women who responded “work for someone else” were classified as employed for wages, and women who responded “self-employed” or stated, “I run my own business” or a similar phrase, were classified as self-employed.

Our outcomes included both physical and mental health outcomes and health behaviors. Physical and mental health outcomes of interest were general health status, body mass index (BMI), obesity, hypertension, diabetes, hyperlipidemia, history of any emotional, nervous, or psychiatric problems, and history of depression. Health behaviors of interest were participation in at least twice-weekly physical activity, binge drinking, and smoking. Determination of health status was based on the survey question, “Would you say your health is excellent, very good, good, fair, or poor?” Respondents who answered “fair” or “poor” were categorized as being in poor health; all other respondents were considered not to be in poor health [12]. BMI and obesity were calculated using self-reported weight and height data. Hypertension, diabetes, hyperlipidemia, history of emotional, nervous, or psychiatric problems, and history of depression were self-reported in response to yes/no survey questions. Participation in at least twice-weekly physical activity was based on responses to survey questions about frequency of engagement in mild, moderate, or vigorous physical activity. Respondents were considered to have demonstrated the health behavior of interest if they answered “more than once a week” or “every day” to any of the three questions. Our binge drinking measure was based on the survey question, “In the last 3 months, on how many days have you had four or more drinks on one occasion?” Respondents who answered one or more days were categorized as having engaged in binge drinking. Finally, our smoking measure was based on “yes/no” responses to the question, “Have you ever smoked cigarettes [> 100 cigarettes in lifetime]?”.

Our covariates were age, education, marital status, number of children residing in the home, hours worked per week, and perceived neighborhood safety (excellent, very good, good, fair, poor). Neighborhood safety was a reverse-coded indicator variable coded as “1” if the neighborhood was rated as unsafe (fair or poor) and coded “0” otherwise.

We constructed three different indicator variables to capture access to health care: health insurance coverage (no health insurance; yes/no), healthcare costs (inability to afford medical care in the last 2 years; yes/no), and healthcare provider access (trouble finding a provider in the last 2 years; yes/no). Each indicator was coded as “1” for the affirmative response and coded as “0” otherwise. These measures, along with education and perceived neighborhood safety (a proxy for socioeconomic status) served to minimize the impact of the potential self-selection of healthier, wealthier women into the self-employment group.

### Statistical analysis

We calculated summary statistics for employment structure, outcomes, and covariates. We then examined the means for continuous variables and the proportions for dichotomous variables, across women employed for wages and self-employed women. All covariates were included in the adjusted analysis. Multivariable linear and logistic regression were used for continuous and dichotomous outcomes, respectively. To determine whether any association between our independent and dependent variables was impacted by healthcare access, we ran additional models controlling separately for each of our three measures of access to care (health insurance coverage, prohibitive care costs, and provider access). All models were weighted using sampling weights provided by HRS. All analyses were conducted using Stata Special Edition 16.1. A *p* value of ≤ 0.05 was used to determine statistical significance for all analyses.

## Results

Of the 4624 working women in our study population, 747 (16%) were self-employed. Table 1 provides unweighted descriptive statistics for outcomes and covariates by employment structure. On average, self-employed women were older than women employed for wages (61.6 vs. 59.1) and worked more hours per week (67.3 vs. 52.0). Self-employed women were more likely not to have health insurance coverage (9.1% vs. 5.3%), more likely to experience prohibitive healthcare costs (8.8% vs. 8.0%), and more likely to have limited healthcare provider access (7.2% vs. 3.7%). In addition, women who were self-employed were less likely to report obesity (31.7% vs. 41.3%) and hypertension (19.1% vs. 27.6%). They were more likely to report participation in at least twice-weekly physical activity (80.4% vs. 72.0%). BMI was on average 27.7 kg/m^2^ for self-employed women and 29.6 kg/m^2^ for women employed for wages. The two groups did not exhibit significant differences in college education, marital status, children residing in the home, perceived neighborhood safety, general health status, diabetes, hyperlipidemia, history of depression, history of emotional, nervous, or psychiatric problems, engagement in binge drinking, or smoking.

**Table 1:** Descriptive Statistics by Employment Type

Patient characteristic or experience, mean or %	Self-employed (n = 747)	Employed for wages (n = 3, 877)	*p*
*Health outcomes*			
Poor health	13.8	14.5	0.40
BMI (kg/m^2^)	27.7	29.6	0.00
Obesity	31.7	41.3	0.00
Hypertension	19.1	27.6	0.00
Diabetes	11.5	14.3	0.07
Hyperlipidemia	31.2	34.4	0.08
Psychological issues	20.0	19.5	0.32
History of depression	25.9	26.8	0.79
*Health behaviors*			
Physical activity > 1×/week	80.4	72.0	0.00
Binge drinking	62.5	66.9	0.33
Smoking	43.8	45.8	0.99
*Covariates*			
Age	61.6	59.1	0.00
College education	8.4	11.5	0.06
Married	65.9	61.2	0.12
Children residing in home	0.45	0.52	0.06
Hours worked per week	67.3	52.0	0.00
Perceived unsafe neighborhood	8.1	8.2	0.03
*Healthcare access controls*			
No health insurance	9.1	5.3	0.00
Prohibitive care costs	8.8	8.0	0.03
Limited provider access	7.2	3.7	0.04

Data is from the University of Michigan Health and Retirement Study (2016 cohort). Bivariates for continuous and dichotomous variables were calculated using the t-test and test of proportions, respectively. A *p* value of ≤ 0.05 was used to determine statistical significance

The first column of Table 2 reports the results of the adjusted analyses, which controlled for age, education, marital status, number of children residing in the home, hours worked per week, and perceived neighborhood safety. Self-employment was found to be associated with a 34% decrease in the odds of reporting obesity (OR = 0.66; 95% CI 0.47, 0.92; *p* = 0.02), a 43% decrease in the odds of reporting hypertension (OR = 0.57; 95% CI 0.41, 0.77; *p* = 0.00), a 30% decrease in the odds of reporting diabetes (OR = 0.70; 95% CI 0.51, 0.96; *p* = 0.03), and a 68% increase in the odds of reporting participation in at least twice-weekly physical activity (RR = 1.68; 95% CI 1.26, 2.23; *p* = 0.00). On average, BMI for self-employed women was 1.79 units lower than it was for women employed for wages (β = − 1.79; 95% CI − 2.56, − 1.03; *p* = 0.00). All other measures were not statistically significant.

**Table 2:** Self-employment and self-reported health outcomes and behaviors, with and without controls for healthcare access

	Adjusted		Controlled for health insurance coverage		Controlled for prohibitive care costs		Controlled for provider access	
*Health outcomes or behaviors, OR or β coefficient (95% CI) and p values*								
Poor health	1.00 (0.73,1.37)	0.98	0.97 (0.71,1.32)	0.84	0.94 (0.70, 1.27)	0.69	0.97 (0.71, 1.33)	0.86
BMI (kg/m^2^)	− 1.79 (− 2.56, − 1.03)	0.00	− 1.78 (− 2.56, − 0.99)	0.00	− 1.85 (− 2.61, − 1.08)	0.00	− 1.81 (− 2.58, − 1.04)	0.00
Obesity	0.66 (0.47, 0.92)	0.02	0.67 (0.47, 0.94)	0.02	0.65 (0.46, 0.91)	0.01	0.66 (0.47, 0.92)	0.02
Hypertension	0.57 (0.41, 0.77)	0.00	0.57 (0.41, 0.78)	0.00	0.56 (0.41, 0.77)	0.00	0.57 (0.42, 0.78)	0.00
Diabetes	0.70 (0.51, 0.96)	0.03	0.70 (0.51, 0.97)	0.03	0.69 (0.50,0.94)	0.02	0.70 (0.51, 0.96)	0.03
Hyperlipidemia	0.85 (0.65, 1.11)	0.22	0.88 (0.68, 1.13)	0.31	0.85 (0.65, 1.10)	0.22	0.85 (0.65, 1.10)	0.21
Psychological issues	1.11 (0.83, 1.50)	0.47	1.10 (0.82, 1.49)	0.52	1.09 (0.81,1.47)	0.58	1.07 (0.79, 1.45)	0.68
History of depression	1.04 (0.83, 1.30)	0.73	1.03 (0.82, 1.29)	0.79	1.01 (0.81, 1.28)	0.90	1.02 (0.81, 1.29)	0.84
Physical activity > 1×/week	1.68 (1.26, 2.23)	0.00	1.69 (1.26, 2.26)	0.00	1.68 (1.26, 2.25)	0.00	1.67 (1.25, 2.23)	0.00
Binge drinking	0.84 (0.67, 1.05)	0.13	0.83 (0.67, 1.04)	0.11	0.83 (0.67, 1.04)	0.11	0.84 (0.67, 1.05)	0.12
Smoking	1.01 (0.57, 1.77)	0.98	1.00 (0.57, 1.77)	0.99	1.00 (0.55, 1.80)	1.00	1.02 (0.58, 1.78)	0.95

Data is from the University of Michigan Health and Retirement Study (2016 cohort). Multivariable linear and logistic regression were used to examine continuous and dichotomous outcomes, respectively. All models controlled for age, college education, marital status, children residing in the home, hours worked per week, and perceived neighborhood safety. Column 1 shows data from the initial multivariate and logistic regressions. Columns 2, 3, and 4 show our outcomes after controlling for each of our three healthcare access measures. All models were weighted for complex survey design and nonresponse. Results are presented as odds ratios, with the exception of BMI, which is presented as a β coefficient. A *p* value of ≤ 0.05 was used to determine statistical significance for all analyses

In columns 2–4 of Table 2 we present the results from the models controlling for our healthcare access variables. The model results did not substantively differ from the main results. When we controlled for health insurance coverage, self-employment was found to be associated with a 33% decrease in the odds of reporting obesity (OR = 0.67; 95% CI 0.47, 0.94; *p* = 0.02), a 43% decrease in the odds of reporting hypertension (OR = 0.57; 95% CI 0.41, 0.78; *p* = 0.00), a 30% decrease in the odds of reporting diabetes (OR = 0.70; 95% CI 0.51, 0.97; *p* = 0.03), and a 69% increase in the odds of reporting participation in at least twice-weekly physical activity (RR = 1.69; 95% CI 1.26, 2.26; *p* = 0.00). BMI for self-employed women was 1.78 units lower than it was for women employed for wages (β = − 1.78; 95% CI − 2.56, − 0.99; *p* = 0.00). Results were similar when we controlled for prohibitive care costs and provider access.

## Discussion

We conducted a weighted cross-sectional analysis to explore the relationship between self-employment and women’s cardiovascular health. We ran additional models to assess the impact, if any, of healthcare access on this relationship. As predicted, our findings showed that self-employed women had significantly better outcomes for several cardiovascular disease risk factors (obesity, hypertension, diabetes, participation in at least twice-weekly physical activity and BMI) as compared to women employed for wages. These results were not found to be impacted by access to health care, which is consistent with our other hypothesis suggesting that factors outside of healthcare access underlie these observed relationships. Notably, there was a large difference in reported engagement in regular physical activity between the two groups. One potential explanation for this observed relationship may be increased schedule flexibility associated with self-employment. Reduced stress, along with consistent physical activity, may also contribute to the observed improvements in obesity, hypertension, diabetes and BMI among self-employed women.

We did not find a significant relationship between self-employment and measures of mental health. This may have been a consequence of the sensitive, but non-specific nature of our mental health measures (history of any emotional, nervous, or psychiatric problems, and history of depression). Studies that employ mental health measures assessed within a timeframe on the scale of days or weeks may yield more information [20]. Narain and Jeffers found positive mental health outcomes for self-employed black women when using “number of self-reported poor mental health days in the last 30 days” as a mental health measure [12].

To our knowledge, this is the first study to examine the relationship between self-employment and a broad range of cardiovascular disease risk factors, health outcomes, and health behaviors in women. It is also the only study on this topic to control for hours worked per week and perceived neighborhood safety, and one of only a few studies to examine the role of healthcare access. Our findings are mostly consistent with those of Yoon and Bernell, who also found that self-employed individuals were more likely to participate in physical activity and have normal-weight and were less likely to report hypertension and diabetes [11]. However, unlike Yoon and Bernell we only find an insignificant negative trend between self-employment and hyperlipidemia [11]. Our findings are also consistent with the work of Narain and Jeffers, who found an association between self-employment and reduced reports of hypertension and “no exercise” among black women [12].

Although this study makes several contributions to the literature, it has some important limitations. Firstly, we were unable to account for selection bias and reverse causality with this particular study design. It may be the case that self-employed women are healthier at baseline than women employed for wages and self-select into a non-traditional employment structure [21]. However, we do attempt to moderate the influence of selection bias by controlling for different proxies of socioeconomic status (education, neighborhood quality, and access to healthcare).

Because of the nature of our dataset, we were also unable to account for some potential confounding variables. Race was not controlled for due to the high prevalence of non-responders to the racial demographics question within the survey. However, perceived neighborhood safety served as a reasonable proxy for race given that perceptions of neighborhood safety tend to vary significantly among racial groups [22]. Additionally, our results are consistent with similar research conducted among black women, so it is unlikely that the exclusion of race as a covariate significantly altered our results [12]. The association between self-employment and health outcomes may also have been impacted by household income, another covariate we did not include in our study due to a high proportion of missing data. However, we controlled for several proxies for income such as education level, perceived neighborhood safety, and healthcare access. Like many other studies investigating the relationship between self-employment and health, we were unable to control for employment industry, which may vary across employment structure and may be associated with our outcomes. However, this issue may not be as problematic in the context of this particular study, given the high level of gender segregation across industries.

We were also unable to distinguish between women who chose self-employment out of business interest (“opportunity” self-employment) and women who may have been pushed into self-employment due to unemployment, or other less favorable factors (“necessity” self-employment). The factors that drive women to pursue self-employment may have appreciable implications for health outcomes. Studies have suggested that both the physical and mental health benefits of self-employment extend to opportunity entrepreneurs, while necessity entrepreneurs may glean only mental health benefits [20]. Additionally, we were not able to control for job description but educational status may partially serve as a proxy for this.

Lastly, our dataset necessitated the use of self-reported health outcomes rather than measured outcomes. Controlling for healthcare access was our main approach to minimizing bias stemming from the use of self-reported measures, which have been shown to be most reliable among individuals with regular access to health care [12].

Our findings suggest that employment structure may have meaningful implications for cardiovascular disease risk factors among women. Consequently, bolstering entrepreneurship among women may be a matter of health in addition to serving as a step towards gender equity. In 2019, self-employed men outnumbered self-employed women by more than two million [23]. Much of the explanation for this disparity has been attributed to personality differences; namely, that women are less inclined towards “entrepreneurial behavior” than men [24]. However, studies have also found that women, regardless of employment structure, are more likely to work part-time due to domestic constraints; this may make maintaining a small business more difficult and push women towards wage work [25]. Women are also more likely to pursue careers in service industries (e.g., healthcare and education), which may be less easily adapted to self-employment [25, 26]. Finally, women generally have less social capital and fewer business connections than men, which may render self-employment a riskier option than wage work [26]. Considering these factors, encouraging women to pursue self-employment may require advocating for more progressive gender attitudes, recruiting more women for business education, and government-assisted economic development [27]. Additionally, supporting women-owned businesses is imperative. Recent literature has revealed that self-employed women have been disproportionately affected by the COVID-19 pandemic [28]. Ensuring that these businesses receive equitable access to resources to keep them afloat during the pandemic and subsequent recovery may be a matter of both economic security and health in both the short and long terms.

While it is not realistic to expect that all women will become self-employed, it may be worth considering how some of the positive features of self-employment such as increased autonomy and flexibility and less exposure to discrimination may be imported into the wage employment context. Of note, the disruption to workplace norms caused by the COVID-19 pandemic poses a unique opportunity to rethink workplace culture. Flex-time is used by some employers to promote employee autonomy, and allows employees some measure of control in their work schedule [29]. Flex-time is generally viewed favorably by employees and has been associated with increased motivation, productivity, job satisfaction, health, and well-being [29, 30]. However, a survey conducted by the Society for Human Resource Management found that only 57% of organizations offered flexible schedules in 2019 [31]. Implementing well-structured flex-time policies could ultimately result in improvements in both mental and physical health. To make flex-time more broadly available, employers would need to adopt technologies that facilitate communication among employees, be transparent about their specific policies, and allow for increased inter-department coordination [31]. There is also a need to implement effective practices to reduce workplace gender-based discrimination. Strategies to reduce discrimination may include deliberate construction of mixed-gender work environments, formalization of decision-making, networking and mentoring programs, increased workplace democracy, and stricter consequences for acts of discrimination [19, 32].

Increasing self-employment among women may be more than an economic or gender-equity issue. This study and other early research suggest that self-employment may be a consequential factor in women’s cardiovascular health. Considering the high burden of CVD among women, it may ultimately be necessary to frame enhanced workplace autonomy, flexibility, and inclusivity as workplace wellness strategies rather than as recruitment or retention tactics.

## Conclusion

Using a cross-sectional study design and HRS data we conducted one of the first studies to explore the relationship between self-employment and risk factors for cardiovascular disease among women. We found that self-employment was negatively associated with several of these risk factors and that this relationship was not likely driven by differences in access to health care, across employment status. Given that cardiovascular disease is the number one cause of death for women, future studies should explore the causal nature of the relationship between self-employment and cardiovascular health in this population.

## Data Availability

The 2016 data analyzed is currently available at the University of Michigan Health and Retirement Study (HRS) site https://hrsdata.isr.umich.edu/data-products/2016-hrs-core.

## Abbreviations

CVD: Cardiovascular disease
HRS: University of Michigan Health and Retirement Study
BMI: Body mass index
